# Clinical and Prognostic Value of *PPIA*, *SQSTM1*, and CCL20 in Hepatocellular Carcinoma Patients by Single-Cell Transcriptome Analysis

**DOI:** 10.3390/cells11193078

**Published:** 2022-09-30

**Authors:** Lisha Mou, Chenyang Jia, Zijing Wu, Boyang Xin, Carmen Alicia Liang Zhen, Bailiang Wang, Yong Ni, Zuhui Pu

**Affiliations:** 1 Department of Hepatopancreatobiliary Surgery, Shenzhen Institute of Translational Medicine, Health Science Center, The First Affiliated Hospital of Shenzhen University, Shenzhen Second People’s Hospital, Shenzhen 518035, China; 2 Imaging Department, Shenzhen Institute of Translational Medicine, Health Science Center, The First Affiliated Hospital of Shenzhen University, Shenzhen Second People’s Hospital, Shenzhen 518035, China

**Keywords:** hepatocellular carcinoma, HCC, scRNA-seq, single cell, cytokine signaling in immune, prediction model, immune microenvironment, TMB, GSEA

## Abstract

Hepatocellular carcinoma (HCC) is the most malignant and poor-prognosis subtype of primary liver cancer. The scRNA-seq approach provides unique insight into tumor cell behavior at the single-cell level. Cytokine signaling in the immune system plays an important role in tumorigenesis and has both pro-tumorigenic and anti-tumorigenic functions. A biomarker of cytokine signaling in immune-related genes (CSIRG) is urgently required to assess HCC patient diagnosis and treatment. By analyzing the expression profiles of HCC single cells, TCGA, and ICGC data, we discovered that three important CSIRG (*PPIA*, *SQSTM1*, and *CCL20*) were linked to the overall survival of HCC patients. Cancer status and three hub CSIRG were taken into account while creating a risk nomogram. The nomogram had a high level of predictability and accuracy. Based on the CSIRG risk score, a distinct pattern of somatic tumor mutational burden (TMB) was detected between the two groups. The enrichment of the pyrimidine metabolism pathway, purine metabolism pathway, and lysosome pathway in HCC was linked to the CSIRG high-risk scores. Overall, scRNA-seq and bulk RNA-seq were used to create a strong CSIRG signature for HCC diagnosis.

## 1. Introduction

According to the most recent data from the International Agency for Research on Cancer, liver carcinoma is one of the most prevalent malignancies globally [1]. In recent years, tremendous progress has been made in diagnosing and treating hepatocellular carcinoma (HCC), the most common type of liver carcinoma [2]. The most effective treatment for early HCC is surgical resection. However, most patients are diagnosed in their late stages and are then unable to seek this treatment [3]. As a result, HCC may not be discovered for a number of years after its onset, usually in its advanced stages [4]. As a result, the diagnostic delay is a crucial factor that affects the overall controllability of HCC. Finding patients at high risk is a crucial step in the prediction of HCC. The gene signature produced from the high-risk group may be used to conduct a risk assessment and to enhance early cancer screening, early diagnosis, and treatment. Various biochemical clinical indicators have been used to screen and evaluate HCC patients. Alpha fetoprotein (AFP) is the most classic one among them. According to the Guidelines for the Diagnosis and Treatment of Primary Liver Cancer (2022 edition), patients are highly suspected to suffer HCC if their serum AFP level is greater than the cutoff value of 400 ng/mL, even in the absence of imaging evidence [5]. PIVKA-II (prothrombin induced by vitamin K deficiency or antagonist-II) is another highly specific biomarker of HCC that was first reported by Liebman in 1984 [6]. PIVKA-II has been included in the guidelines of the Asian Pacific Association of Liver Diseases and the Japan Association of Liver Diseases and is recommended for use in high-risk population screening, auxiliary diagnosis, monitoring treatment effect, and as a predictor of prognosis and recurrence. Several studies have indicated that PIVKA-II may be a convincing supplement to AFP in the clinical diagnosis of HCC [7,8,9].

Cells of the immune system produce cytokines, including lymphocytes, monocytes, macrophages, B cells, and T cells [10]. Cytokines are molecular messengers that allow immune system cells to communicate to generate coordinated, effective, yet self-limiting antigen responses. Although many forms of immune system communication occur via direct intercellular connections, cytokine production enables a more diverse and efficient dissemination of immunological signals [11]. The use of cytokines in the treatment of multiple tumors is essential, including HCC, melanoma, renal cell carcinoma, etc. [12,13]. At the tumor site, cytokines stimulate immunological effector cells, thereby enhancing the identification of tumor cells. Interferons and interleukins have a relationship with enhanced immunostimulatory effects [10]. As a result, numerous cancer treatments based on cytokines or cytokine antagonists have been created [14]. However, cytokine-based therapy showed limited therapeutic efficacy for patients with advanced-stage disease.

With the development of biotherapy and precise treatment, RNA sequencing can explore evidence at the genetic level to guide clinical decisions. Previous studies based on bulk RNA sequencing have established multiple gene signatures to predict the prognosis of HCC patients [15,16,17], but these signatures have not been used in the clinic. In bulk RNA sequencing, RNA signals from diverse cells within a sample are mixed to represent the average RNA content of a sample. Therefore, cell type prevalence has a significant influence on it. However, there are distinct genetic characteristics associated with HCC that can differentially promote its progression. Therefore, bulk RNA sequencing cannot study rare or heterogeneous cell populations. In contrast to bulk-RNA sequencing analysis, single-cell RNA sequencing (scRNA-seq) allows the study of transcriptional activity within an individual cell and enables the discovery of the gene expression of small but clinically significant tumor subpopulations.

The TMB concept first came into view as an emerging tumor immunotherapy biomarker in 2014. Along with the approval of pembrolizumab used for the treatment of TMB-high (≥10 mutations/Mb) solid tumors, TMB was approved by the FDA as a tumor biomarker in 2020 [18]. A large sample analysis that contained various kinds of cancer showed that 83% of MSI-H samples also had TMB-H (97% of them had TMB ≥ 10 mutations/Mb). However, in contrast, only 16% of TMB-H samples were classified as MSI-H [19]. This may indicate that TMB has a wider range of application. Other evidence also showed that TMB had a predictive effect on immunotherapy in a variety of tumors [20,21]. Encouragingly, recent studies have proven that TMB in plasma can be accurately and repeatedly measured and correlated with immune checkpoint inhibitor efficacy [22]. This confirmed the effectiveness of blood TMB in predicting the efficacy of immunotherapies, which may be good news for those who had difficult biopsies.

Patients with HCC metastasis usually have a poor prognosis, so the differential genes identified from patients with HCC metastasis versus patients with primary HCC may help to develop markers for predicting poor prognosis. To find prospective signatures using single-cell RNA sequencing data, we studied primary and metastatic HCC samples. We created a three-gene prognostic signature that includes *PPIA*, *SQSTM1*, and *CCL20*. The ICGC dataset provided validation for the signature. Moreover, a predictive nomogram was established and evaluated. The prognostic status of patients with HCC may be guided by the signature and nomogram created in this study.

## 2. Materials and Methods

### 2.1. Data Collection and Preparation

The single-cell RNA sequencing (scRNA-seq) datasets of 10 hepatocellular carcinoma (HCC) patients were from GSE149614 [23]. The bulk RNA data of HCC (TCGA-LIHC) were downloaded from The Cancer Genome Atlas (TCGA). Additionally, the ICGC-LIRI-JP dataset was acquired from the International Cancer Genome Consortium database. The complete survival information and expression profiles of HCC patients were enrolled. The TCGA-LIHC dataset included 377 HCC samples, and the ICGC-LIRI-JP datasets included 260 HCC samples. Cytokine signaling in immune-related genes (CSIRG) was obtained from the Reactome Pathway Database.Available online: https://reactome.org/ (accessed on 13 January 2022).

### 2.2. scRNA-seq Data Processing

ScRNA-seq analysis was performed in Seurat (v4.1.0, New York Genome Center, New York, NY, USA), ggplot2, and dplyr. In addition, the scRNA-seq profiles of 10 HCC patients of 56 440 cell samples were collected from GSE149614 [23]. Ten HCC patients were examined through three relevant sites: The primary tumor, the portal vein tumor thrombus, and the metastatic lymph node. The data were then scaled, and principal component analysis (PCA) was computed. The nearest neighbors were then determined, and three-dimensional plot_ly was performed to visualize the outcomes. The findMarkers function in the Seurat package was used for the variance analysis. Differences in gene expression between the two groups (group 1: Primary HCC, group 2: Metastatic HCC) were analyzed using the Wilcoxon rank-sum test.

### 2.3. Identification of Signature

Before conducting the univariate Cox regression analysis, we first adopted the merge function in R studio to integrate the expression profiles of the 26 CSIRG with corresponding survival information in the TCGA-LIHC dataset. Before any subsequent analysis, data collected from TCGA-LIHC and ICGC-LIRI-JP were arranged correspondingly into training and validating groups. Moreover, LASSO Cox analysis was performed, and the CSIRG signature was established using the following equation: Risk score = ∑ (βi × Expi). ROC was further employed to validate the results of the training set. The CSIRG predictive risk score value in the training group was quantified by the equation. Kaplan–Meier and ROC analyses were used to verify the effective prognostic potency of the CSIRG signature in both the training and verification groups. Patients were divided into the CSIRG high- and low-risk groups according to the median risk score. In the TCGA-LIHC and ICGC-LIRI-JP datasets, the median risk scores were 0.90094 and 0.85695, respectively.

### 2.4. Model Construction

The CSIRG signature was combined with all the clinical features obtained from the LIHC for the training set. First, univariate Cox models were used to calculate hazard ratios. Furthermore, multivariable Cox analysis was used to testify to the prognostic independence of the scRNA signature, where *p* < 0.05 was regarded as statistically significant. The area under the curve (AUC) of 1-, 3-, and 5-year OS outcomes was used to compare the prediction accuracy of the new signature. In addition, the decision curve analysis and calibration curve were plotted to validate the constructed model.

### 2.5. Histological Data Analysis of HCC

The protein expression of PPIA, SQSTM1, and CCL20 in HCC was investigated based on immunohistochemistry (IHC) data from the Human Protein Atlas (HPA) database. Available online: http://www.proteinatlas.org (accessed on 4 September 2022). 

### 2.6. Tumor Mutational Burden (TMB)

To calculate the TMB of each LIHC tumor sample, the processed data for somatic mutations were selected using the VarScan platform in the TCGA-LIHC cohort. The R package “maftools” was used to analyze MAF files to find nonsynonymous somatic mutations. Frame shift del, frame shift ins, splice site, translation start site, nonsense mutation, nonstop mutation, in frame del, in frame ins, and missense mutation are all categorized as nonsynonymous mutations in accordance with the criteria for this package. The gene mutation frequency was analyzed between the CSIRG high-risk and low-risk groups.

### 2.7. Gene set Enrichment Analysis (GSEA)

As described above, we classified the TCGA-LIHC dataset into two groups with high and low CSIRG risk scores. We further performed GSEA to explore the significant pathways enriched in the CSIRG high- and low-risk score groups. The first step was to create a differential analysis of all the genes between the CSIRG high- and low-risk score groups. The genes were then ranked according to the log2-fold change. Then, GSEA was used to examine the signaling pathways associated with the CSIRG high- and low-risk score groups. The important pathways that were enriched in each group were subjected to the normalized enrichment score |NES| ≥ 1 and *p* value < 0.05 tests. GSEA (version 4.0.03) was used for analysis (http://www.broadinstitute.org/gsea/, accessed on 13 January 2022). The molecular signature dataset used for the KEGG pathway was “c2.cp.kegg. v7.5.1.symbols.gmt”. One thousand permutations were used in the GSEA with gene set permutations.

### 2.8. Immune Infiltration

The CSIRG high- and low-risk patients were analyzed using xCell with a cell type enrichment score for immune subset enrichment [24]. The data were downloaded through the xCell website in accordance with previous reports [24]. The xCell results had a *p* < 0.05 value selected. Violin plots were used to visualize the results.

### 2.9. Statistical Analysis

Statistical analyses were performed using R v4.0.5. Moreover, *p* < 0.05 was considered statistically significant.

## 3. Results

### 3.1. Identification of 26 Cytokine Signaling in Immune-Related Genes (CSIRG) in GSE149614

**Figure 1:**
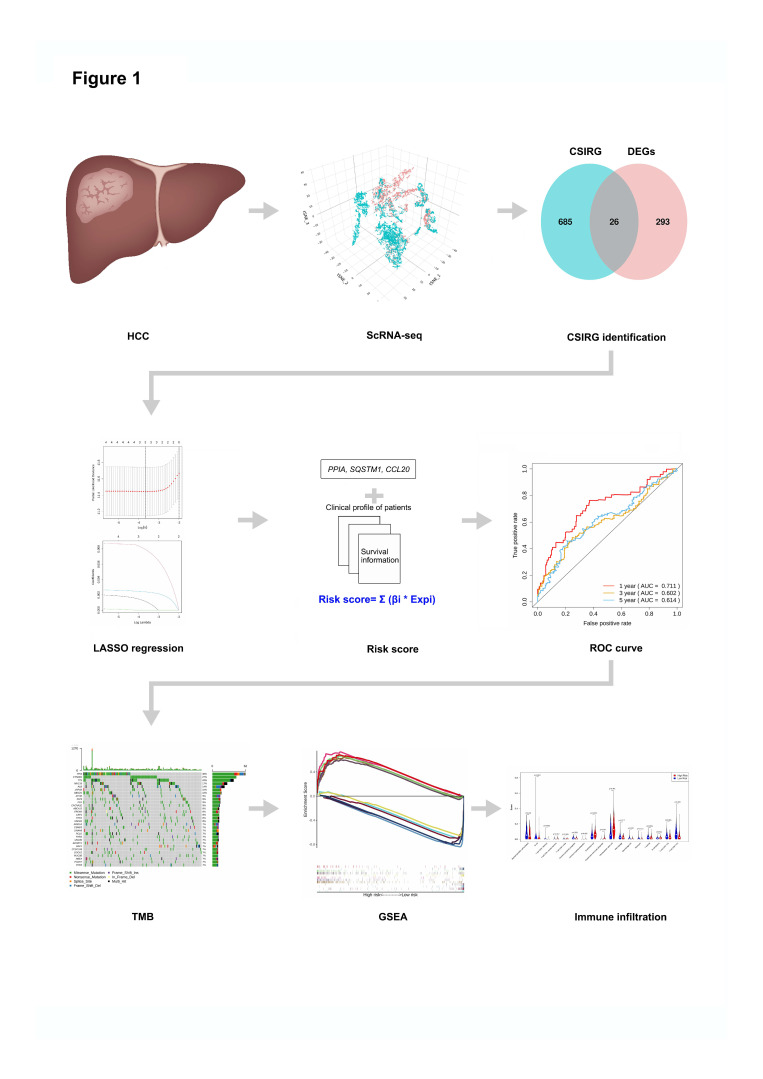
Workflow of this study.

The workflow of this study is shown in Figure 1. We first accessed the single-cell dataset GSE149614 for 10 HCC patients with primary and metastatic samples. Then, 26 differentially expressed cytokine signaling in immune-related genes (CSIRG) between primary and metastatic HCC samples were identified. Next, the expression profiles of the 26 CSIRG with corresponding survival information from the TCGA-LIHC cohort were used for LASSO Cox regression analysis. Three important genes (*PPIA*, *SQSTM1*, and *CCL20*) were identified to calculate the risk score of each patient and construct the prediction model. The performance of the prediction model was evaluated by ROC curves. Then, patients in TCGA-LIHC were separated into two groups based on the median risk score. Finally, tumor mutational burden TMB analysis, gene set enrichment analysis (GSEA), and immune infiltration analysis were performed to compare the CSIRG high- and low-risk groups of patients.

**Figure 2:**
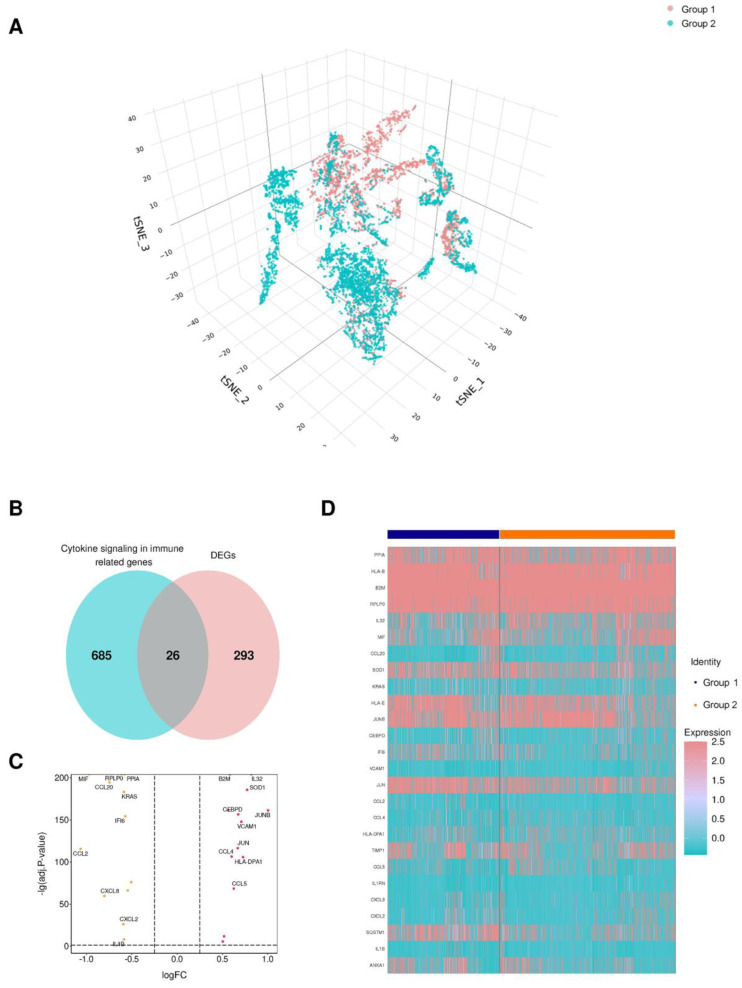
Identification of cytokine signaling in immune-related genes (CSIRG) in the GSE149614 dataset. (**A**) Nonlinear dimensionality reduction by the t-SNE algorithm. (**B**) Differentially expressed genes and CSIRG from Reactome. (**C**) A volcano map involving the up- and downregulated CSIRG. (**D**) The expression of 26 CSIRG in two cell populations. The primary HCC tissue was denoted as group 1, and metastatic HCC tissue was denoted as group 2.

Single-cell transcriptome analysis was utilized in this study to understand the genes that are differentially expressed between primary hepatocellular carcinoma (HCC) and metastatic HCC using the GSE149614 dataset [23]. By performing nonlinear dimensionality reduction via the t-SNE algorithm, samples from the GSE149614 dataset were clustered, as shown in Figure 2A. Overall, differentially expressed genes were identified between two cell subpopulations: Primary HCC and metastatic HCC. These differentially expressed genes intersected with the cytokine signaling in immune-related genes from the Reactome Pathway Database (https://reactome.org/), and 26 genes were cytokine signaling in immune-related genes (Figure 2B, Supplementary Table S1). As shown in the volcano plots, 14 were upregulated, while 12 were downregulated (Figure 2C). Moreover, the heatmap shows the expression of the 26 CSIRG in the two cell subpopulations (Figure 2D).

### 3.2. Construction of a Three-Gene Signature Based on PPIA, SQSTM1, and CCL20

**Figure 3:**
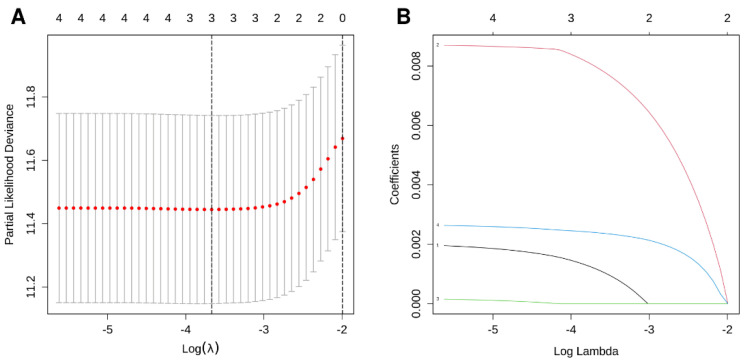
LASSO regression results. (**A**) LASSO coefficient profile of four overall survival (OS)-related CSIRG. (**B**) LASSO deviation profile of four OS-related CSIRG.

**Figure 4:**
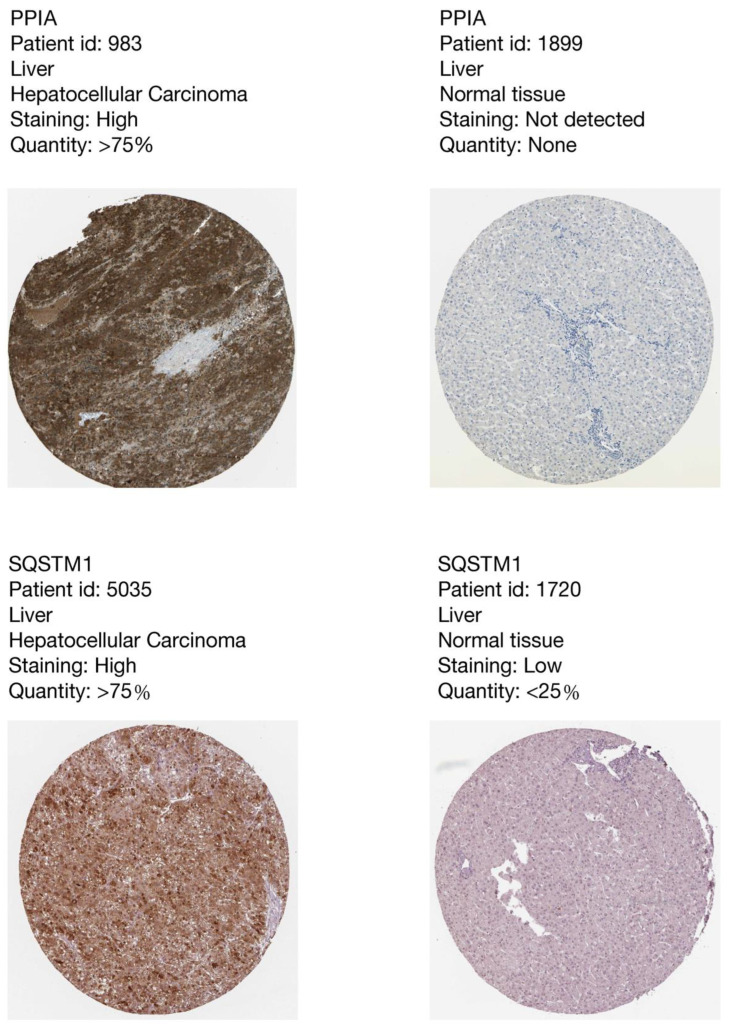
The immunohistochemistry results of PPIA and SQSTM1 from the Human Protein Atlas showed higher expression levels of PPIA and SQSTM1 in HCC tissues than in normal tissues.

**Figure 5:**
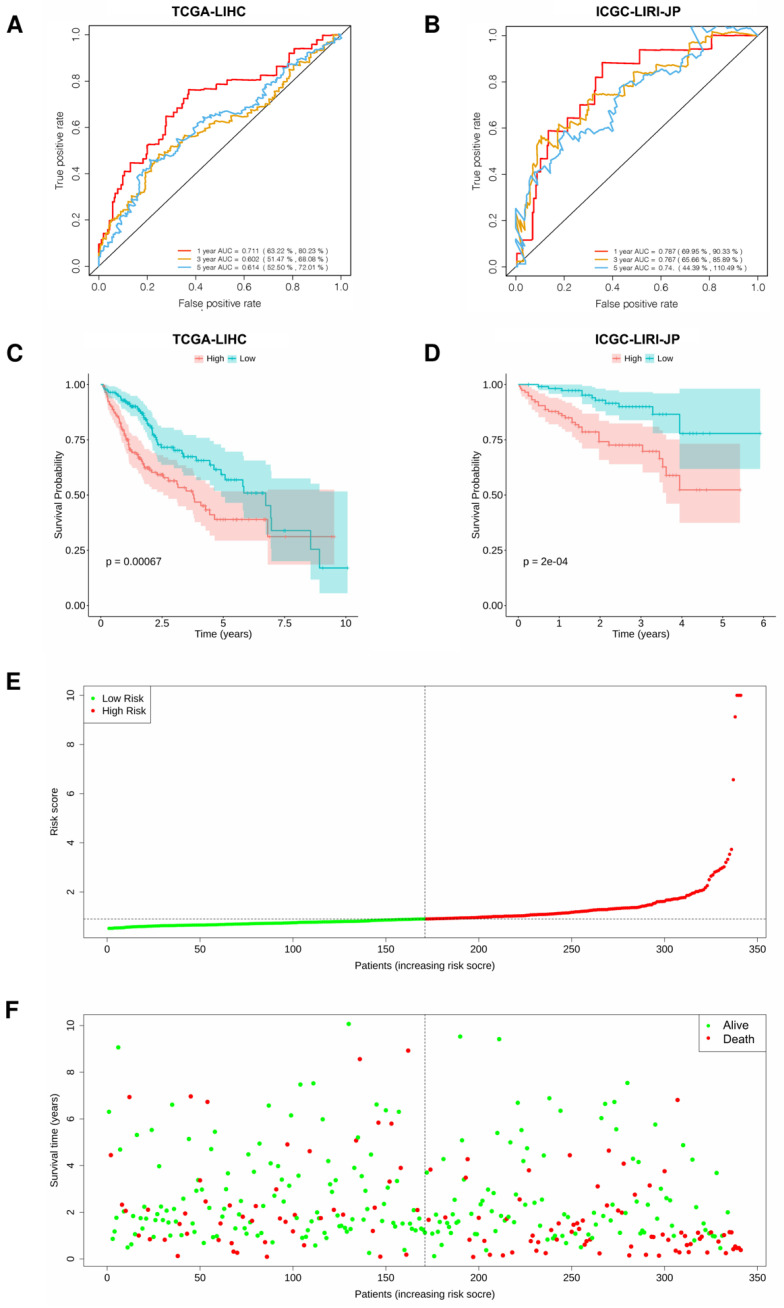
Validation of the three-gene signature in HCC. (**A**,**B**) Time-dependent ROC curves of the three-gene signature in two datasets. (**C**,**D**) Kaplan-Meier curves of risk subgroups in the two datasets. (**E**,**F**) The association between survival status and RS. Integration nomogram based on risk score and cancer status for HCC prediction.

We used the 26 CSIRG genes found in the single-cell analysis as the initial gene list for univariate Cox regression analysis. Before the univariate Cox regression analysis, we first integrated the expression profiles of the 26 CSIRG with corresponding survival information in the TCGA-LIHC dataset. Four overall survival (OS)-related CSIRG (*CCL20*, *PPIA*, *RPLP0*, and *SQSTM1*) were filtered out through univariate Cox regression analysis (Supplementary Table S2). The four CSIRG found in univariate Cox regression analysis were used as the gene initial list for LASSO regression analysis. The LASSO method was then employed, and three genes were identified for further analysis (Figure 3A,B). By using multivariate Cox regression analysis, the selected OS-related CSIRG were analyzed, and all three genes (*PPIA*, *SQSTM1*, and *CCL20*) were identified (Supplementary Table S3). We used IHC data from the Human Protein Atlas (HPA) database to compare the protein levels of PPIA and SQSTM1 in HCC tissues to those in normal tissues. We discovered that the expression of PPIA and SQSTM1 was much higher in HCC tissue than in normal tissues (Figure 4). Since CCL20 data are not available in the HPA database, we did not analyze CCL20. Moreover, the risk score (RS) equation was established based on three genes. The following demonstrates the risk score equation: RS = 0.00951 × *PPIA* + 0.00261 × *SQSTM1* + 0.00213 × *CCL20*. The TCGA-LIHC dataset was separated into a CSIRG low-risk group (*n* = 171) and a CSIRG high-risk group (*n* = 170) based on the median RS (median RS = 0.90094, Supplementary Table S4). In addition, the ICGC-LIRI-JP dataset was separated into two groups based on the median RS (median RS = 0.85695, CSIRG low-risk group, n = 116; CSIRG high-risk group, n = 116). Furthermore, the time-dependent ROC curves verified the performance of the three-gene signature. The TCGA-LIHC dataset had AUC values of 0.711 (1 year), 0.602 (3 years), and 0.614 (5 years), while the ICGC-LIRI-JP dataset had AUC values of 0.787 (1 year), 0.767 (3 years), and 0.74 (5 years) (Figure 5A,B). The three-gene signature was then validated by Kaplan-Meier curves. Both datasets demonstrated the same results and determined that the OS of the CSIRG low-risk groups was better than that of the CSIRG high-risk groups (Figure 5C,D). In addition, Figure 5E,F demonstrate the association between survival status and RS in the TCGA dataset. The survival rates of HCC decreased with increasing RS.

### 3.3. Integration Nomogram Based on RS and Cancer Status

**Figure 6:**
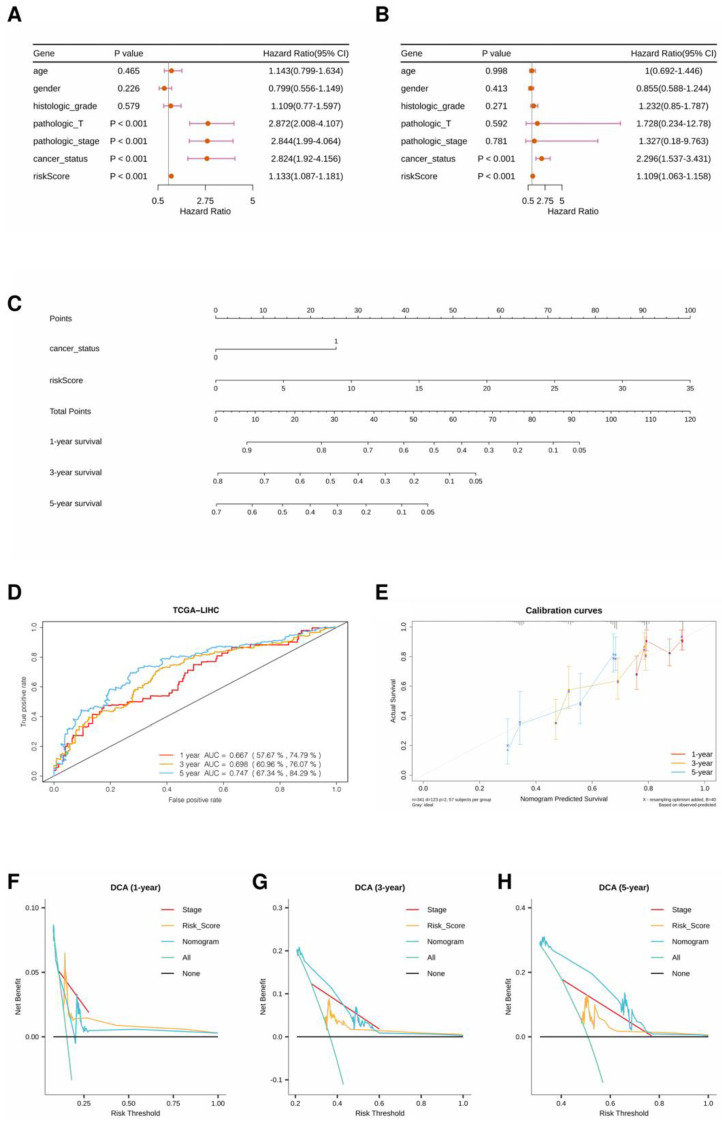
(**A**) Forest plot of univariate regression analysis. (**B**) Forest plot of multivariate regression analysis. (**C**) A nomogram incorporating cancer status and risk score for prognosis. (**D**) ROC curves of the nomogram with AUCs of 0.667, 0.698, and 0.747 at 1, 3, and 5 years, respectively. (**E**) Comparison of predicted and actual OS in the calibration curve. (**F**–**H**) Decision curve analyses of models in the TCGA-LIHC dataset.

The forest plots of the univariate and multivariate Cox regression analyses suggested that cancer status and RS were independent clinical factors related to OS (Figure 6A,B). Then, the generalized linear model (GLM) regression algorithm was used to develop a nomogram incorporating cancer status and RS for HCC prognosis (Figure 6C). The AUCs of the ROC curves for 1-, 3-, and 5-year OS forecasting were 0.667, 0.698, and 0.747, respectively (Figure 6D). The calibration curves indicated that the predicted 1-, 3-, and 5-year OS rates were close to the actual OS rates (Figure 6E). Moreover, the clinical utility of the nomogram was compared with that of the three-gene signature by decision curve analysis. As shown in Figure 6F–H, the nomogram has higher net benefits than the three-gene signature and TNM stage when predicting 3-year and 5-year overall survival.

### 3.4. Comparison of Genomic Mutations in the Three-Gene Signature

**Figure 7:**
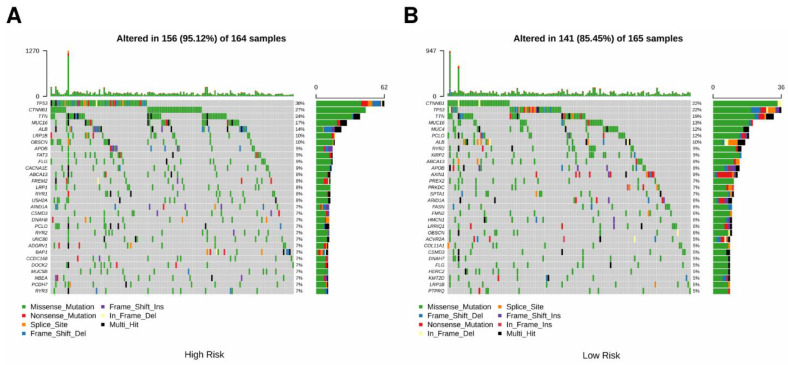
Comparison of genomic mutations between the CSIRG high-risk group (**A**) and the CSIRG low-risk group (**B**) of the TCGA-LIHC dataset.

To investigate the genomic differences between the two risk groups, the genetic mutations of the two groups in the TCGA cohort were analyzed. The tumor mutation profile was matched with RS and presented in the waterfall plot (Figure 7A,B). Most genes had higher mutation rates in the CSIRG high-risk group. Specifically, the mutation rates of *TP53*, *CTNNNB1*, and *TTN* were 38%, 27%, and 24% in the CSIRG high-risk group and 22%, 22%, and 19% in the CSIRG low-risk group, respectively (Figure 7A,B). The clinical information and TMB results of the TCGA cohort are shown in Table 1. In conclusion, the increase in RS may result in an increase in the proportion of mutated genes.

### 3.5. Enrichment Analysis of the Three-Gene Signature

**Figure 8:**
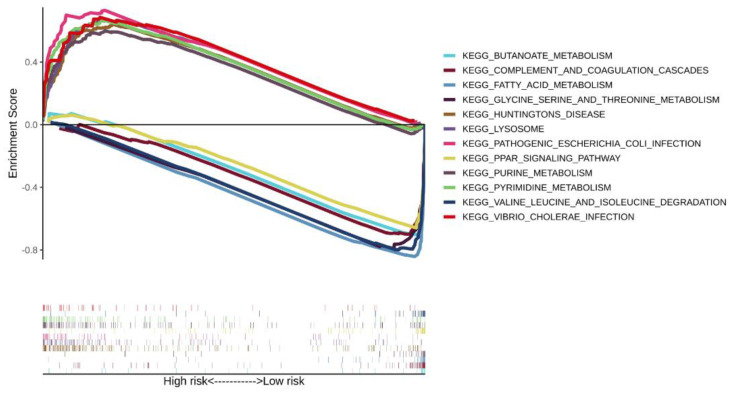
Comparison of enriched signaling pathways between the risk subgroups of the TCGA-LIHC dataset.

The significantly correlated pathways in the CSIRG high- and low-risk groups of the TCGA-LIHC dataset were screened by gene set enrichment analysis. A total of six pathways were selected in the CSIRG high- and low-risk groups (Figure 7). Specifically, pyrimidine metabolism, purine metabolism, lysosome, Huntington’s disease, pathogenic *Escherichia coli* infection, and vibrio cholerae infection were correlated with the CSIRG high-risk group. In contrast, the PPAR signaling pathway, glycine, serine and threonine metabolism, fatty acid metabolism, complement and coagulation cascades, valine, leucine and isoleucine degradation, and butanoate metabolism were correlated with the CSIRG low-risk group (Figure 8 and Supplementary Table S5).

### 3.6. Associations of the Three-Gene Signature with the Immune Microenvironment

**Figure 9:**
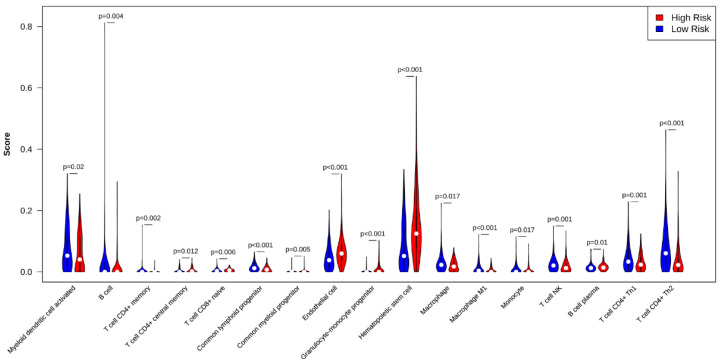
Comparison of tumor-infiltrating immune cells between the risk subgroups of the TCGA-LIHC dataset.

The difference in immune infiltration between the CSIRG high- and low-risk groups was analyzed through xCell algorithms. Specifically, central memory CD4+ T cells, naïve CD8+ T cells, common myeloid progenitors, endothelial cells, granulocyte-monocyte progenitors, hematopoietic stem cells, and plasma B cells were highly distributed in the CSIRG high-risk group. However, activated myeloid dendritic cells, B cells, memory CD4+ T cells, common lymphoid progenitors, macrophages, M1 macrophages, monocytes, NKT cells, Th1 CD4+ T cells, and Th2 CD4+ T cells were highly distributed in the CSIRG low-risk group (*p* value < 0.05) (Figure 9, Supplementary Table S6).

## 4. Discussion

With CSIRG high-throughput scRNA-Seq profiles aiding in the identification of potential biomarkers for predicting patient survival and response to treatment, the pathophysiology, and prognostic variables of HCC (HCC) could be explored. The overall survival probability of HCC patients remains relatively low due to a lack of effective therapy. The expression of cytokines is related to the progression of HCC [25]. As a result, cytokine signaling in immune-related genes (CSIRG) may indicate cancer development and predict HCC patients. The transcription levels of CSIRG in HCC tumor tissues were analyzed in detail, and the relationship among differentially expressed genes, as well as overall survival probability, was established.

As a first step, we used single-cell RNA sequencing and bulk RNA sequencing to find CSIRG that were different in primary and metastatic HCC. Using LASSO regression and DEGs between the two subtypes, we created a three-gene model consisting of *PPIA*, *CCL20*, and *SQSTM1*. Our model showed good performance in both the training cohort and validation cohort. Although it is not common to obtain better results in validation sets than in training sets, our results showed that the AUC results in the ICGC cohort were better than those in the TCGA cohort (Figure 5A,B). Similar results were also reported by previous studies [26]. The reason for better results in the validation cohort may be that the training cohort and validation cohort are independent cohorts.

Several studies have shown that overexpression of *CCL20* increases the migration and proliferation of lung cancer cells through the *PI3K* pathway [27]. Selective recruitment of regulatory T cells (Tregs) by *CCR6* and its ligand *CCL20* in the tumor microenvironment may inhibit the proliferation of *CD4CD25* and INF-γ secretion and promote the development of HCC [28]. In HCC caused by cirrhosis, myofibroblasts oversecrete *CCL20*, which regulates aerobic glycolysis-driven HCC through the *ERK*/*PKM2* pathway [29]. The *SQSTM1* gene causes various carcinomas, including HCC. Its product p62, which acts as an adaptor to degrade molecules through autophagy, provides insight into the effects of *SQSTM1* in various cancers [30]. In breast cancer, the *PPIA*/*CrkII* axis modulates host antitumor immune escape [31]. In HCC, hypoxia increases immune infiltration through the *PPIAP22*/*miR-197-3p*/*PPIA axis* [32].

A nomogram based on the CSIRG risk score was built and verified, and then it was combined with the tumor status of patients with HCC. We identified a positive correlation between genes (*TP53*, *MUC16*, *CTNNB1*, and *TTN*) and HCC occurrence. Among these, *MUC16* is often mutated in different kinds of human cancer and has been linked to increased cancer cell proliferation [33,34,35,36]. Of note, *TTN* mutations are associated with a high tumor mutation load, which is consistent with our study. TMB has recently been established as an effective and new biomarker for predicting immunotherapy response in a range of cancers. Additionally, we found that the tumor mutation burden (TMB) in the CSIRG high-risk group was higher than that in the CSIRG low-risk group. Using the xCell algorithm, we were able to quantify the condition of immune infiltration. In our results, central memory CD^4+^ T cells, naïve CD^8+^ T cells, common myeloid progenitors, endothelial cells, granulocyte-monocyte progenitors, hematopoietic stem cells, and plasma B cells were significantly correlated with the CSIRG high-risk group. Among these, our study discovered the association of common myeloid progenitor and granulocyte-monocyte progenitor with HCC CSIRG high-risk groups for the first time. Regulatory T cells (Tregs) are highly correlated with tumor tissue and can suppress antitumor immune responses [28]. These findings provide a complete picture of how and why CSIRG might play a role in the development and progression of HCC. We conducted functional enrichment analysis of several common biological pathways to further prove the validity of cytokine signaling in immune-related genes in HCC. Consistent with other studies, pyrimidine metabolism, purine metabolism, and fatty acid metabolism are highly enriched and are vital in tumor progression [37,38,39,40].

There are several limitations to this study. First, compared with large-scale bulk-RNA sequencing projects such as TCGA, which have 377 HCC patient samples, the patients of scRNA-seq datasets in this study only include a small number of patients (only 10 patients). However, as mentioned in the manuscript, our single-cell dataset includes 56,440 cell samples, which means many more samples than TCGA datasets, and much more information may be provided. Since the immune profile in the tumor microenvironment and correspondingly the landscape of cytokine signaling in immune-related genes may be more heterogenous, validation of our model with larger sample size is our direction for future improvement. Second, the absence of a real-world sample to validate our prediction model is also a limitation of this study.

Overall, we used scRNA-seq and bulk RNA-seq to create CSIRG signatures that were then verified in patients. The CSIRG signature has potential predictive value for HCC, and further study on the mechanism is recommended.

## Figures and Tables

**Table 1:** Clinical information for TCGA cohort.

Characteristics	High (N = 151)	Low (N = 153)	Overall (N = 304)
**Age**			
<60	73 (48.3%)	74 (48.4%)	147 (48.4%)
≥60	78 (51.7%)	79 (51.6%)	157 (51.6%)
**Gender**			
Female	45 (29.8%)	48 (31.4%)	93 (30.6%)
Male	106 (70.2%)	105 (68.6%)	211 (69.4%)
**Histologic_grade**			
G1	16 (10.6%)	27 (17.6%)	43 (14.1%)
G2	66 (43.7%)	79 (51.6%)	145 (47.7%)
G3	61 (40.4%)	43 (28.1%)	104 (34.2%)
G4	8 (5.3%)	4 (2.6%)	12 (3.9%)
**Pathologic_T stage**			
T1	57 (37.7%)	95 (62.1%)	152 (50.0%)
T2	44 (29.1%)	30 (19.6%)	74 (24.3%)
T3	43 (28.5%)	25 (16.3%)	68 (22.4%)
T4	7 (4.6%)	3 (2.0%)	10 (3.3%)
**Pathologic_stage**			
Stage I	56 (37.1%)	95 (62.1%)	151 (49.7%)
Stage II	43 (28.5%)	30 (19.6%)	73 (24.0%)
Stage III	50 (33.1%)	28 (18.3%)	78 (25.7%)
Stage IV	2 (1.3%)	0 (0%)	2 (0.7%)
**Cancer_status**			
Tumor free	75 (49.7%)	96 (62.7%)	171 (56.3%)
With tumor	76 (50.3%)	57 (37.3%)	133 (43.8%)
**TMB**			
Average	3.9	3.3	3.6

## Data Availability

GSE149614: https://www.ncbi.nlm.nih.gov/geo/query/acc.cgi?acc=GSE149614; (accessed on 13 January 2022); TCGA-LIHC: https://portal.gdc.cancer.gov/projects/TCGA-LIHC; (access on 13 January 2022). ICGC-LIRI-JP: https://dcc.icgc.org/projects/LIRI-JP; (accessed on 13 January 2022). Human Protein Atlas (HPA) database: http://www.proteinatlas.org; (accessed on 4 September 2022). Cytokine signaling in immune-related genes (CSIRG) was obtained from the Reactome Pathway Database (https://reactome.org/). (accessed on 13 January 2022).

## Supplementary Materials

The following supporting information can be downloaded: https://www.mdpi.com/article/10.3390/cells11193078/s1, Table S1: Differentially expressed cytokine signaling in immune-related genes between primary and metastatic HCC; Table S2: Univariate Cox regression analysis results; Table S3: Multivariate Cox regression analysis results; Table S4: Risk score (RS) of each patient; Table S5: GSEA results; Table S6: XCELL results.
